# Granulomatous reaction to migrating silicone oil clinically mimicking a xanthelasma: A case report

**DOI:** 10.1016/j.ijscr.2020.04.071

**Published:** 2020-05-07

**Authors:** Saleh Hamad Alrashed, Hind Manaa Alkatan, Adel H. Alsuhaibani

**Affiliations:** a Department of Ophthalmology, College of Medicine, King Saud University, Riyadh, Saudi Arabia; b Department of Pathology, College of Medicine, King Saud University, Riyadh, Saudi Arabia

**Keywords:** Silicone oil migration, Xanthelasma, Pseudo-xanthelasma, Case report, Granuloma

## Abstract

**Introduction:**

Silicone oil has been used for many years in retinal surgeries for retinal detachment. One of its reported complications is oil migration to the periorbital area, resulting in granulomatous reaction.

**Presentation of case:**

A 56-year-old lady, with history of retinal detachment that was repaired by vitrectomy, silicone oil removal and epi-retinal membrane peeling, presented to us with unilateral ptosis and a skin lesion that resembled xanthelasma.

**Discussion:**

Histopathology of this lesion showed silicone oil infiltrating the surrounding connective tissue and fat with absence of foamy histiocytes.

**Conclusion:**

We are reporting a case of silicone oil migration with pseudo-xanthelasma lesion. This has been reported only twice to the best of our knowledge in the English-written literature.

## Highlights

• Distant silicone oil migration may occur following retina surgery.
• Silicone oil in periocular tissue results in granulomatous reaction.
• It may resemble a periocular xanthelasma-like skin lesion.
• Tissue diagnosis is essential for confirmation in such cases.

## 1 Introduction

Silicone oil has been used for many years in retinal surgeries for retinal detachment. Silicone oil has its complications, and silicone oil migration has been reported several times in the literature [[1], [2], [3], [4], [5], [6], [7], [8], [9], [10], [11]]. This infiltration often results in extensive spread with intense inflammatory response. This inflammation may weaken the levator muscle, and cause ptosis [12]. We report a case of silicone oil migration causing ptosis and xanthelasma-like picture. Other than our case to the best of our knowledge, his has been only reported twice in the English-written literature [6,11].

## 2 Presentation of case

A 56-year-old lady, who is medically free presented to our hospital in December 2018 with the complaint of a drooping right upper eyelid, following a recent retina procedure elsewhere. She had undergone five retinal surgeries because of recurrent retinal detachment, but her last surgery involved epi-retinal membrane peeling, silicone oil removal, and peeling of the internal limiting membrane two months earlier.

**Fig. 1:**
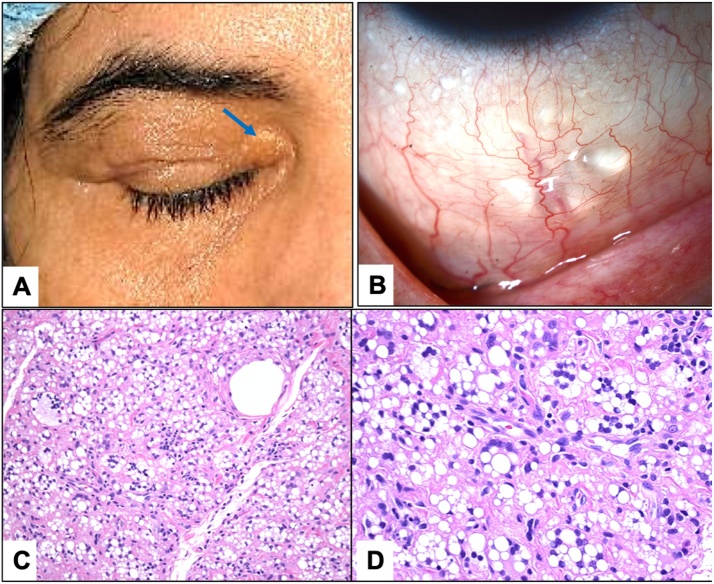
**A:** The clinical appearance of the right upper eyelid masses with a yellowish flat xanthelasma-like area near the medial canthus (Blue arrow). **B:** Subconjunctival collection of silicone oil in the right eye inferior fornix. **C:** The histopathological appearance of the skin in the same area showing the empty silicone oil round spaces surrounded by epithelioid cells and giant cells (Original magnification X200 Hematoxylin and eosin). **D:** Higher power of the silicone oil and the surrounding granulomatous reaction (Original magnification X400 Hematoxylin and eosin). (For interpretation of the references to colour in this figure legend, the reader is referred to the web version of this article).

The patient’s visual acuity (VA) of her right eye was hand motion, while it measured 20/40 in her left eye. The intraocular pressure (IOP) measured 21 and 20 mmHg in her right and left eye respectively. The extraocular motility (EOM) was normal on both sides. She had 2 mm of right upper eyelid ptosis, with a superficial scar along the skin of her right upper lid crease. Multiple slightly raised yellowish skin masses were noted on her upper eyelid, which we thought to be due to migrating silicone oil. xanthelasma-like lesion was also noted in the medial canthal region (Fig. 1a). Multiple pockets of silicone oil were also observed in the subconjunctival/sub-tenon area in all 4 quadrants of the conjunctival fornices (Fig. 1b). She had a clear cornea, and deep/quite anterior chambers in both eyes. She was also observed to have a stable posterior chamber intraocular lens (IOL) in her right eye, and immature cataract in the left. The retina was flat in both eyes. She underwent surgical ptosis repair, combined with upper eyelid blepharoplasty, and excisional biopsy of the xanthelasma-like lesion from the upper lid in September 1, 2019.

Histopathology of the excised skin showed marked fibrosis with several empty spaces representing pools of silicone oil. The silicone oil infiltrated the surrounding connective tissue and fat. In many areas, surrounding numerous epithelioid cells representing ill-defined granulomas was present in addition to other chronic inflammatory cells: lymphocytes and plasma cells (Fig. 1c & d). However, there were no foamy histiocytes to support the diagnosis of xanthelasma even in the areas that were clinically identified as such.

In the last follow-up of the patient in February 9, 2020, she had a good outcome and mild local scarring. Information was obtained and reported in a manner that was compliant with the standards set forth by the Health Insurance Portability and Accountability Act and the Declaration of Helsinki as amended in 2013. General informed written consent was obtained from the patient including anonymous use of information for presentations and contributions.

## 3 Discussion

Silicone oil has been used in retinal surgery for retinal detachment with the following possible complications: cataract, glaucoma, corneal edema and retinal toxicity. Silicone oil migration has been reported in the literature. Several theories have been proposed for silicone oil migration. The most agreed upon was silicone oil migration though the sclerotomy wound. Therefore, proper closure of the sclerotomy wound – with careful removal of any excess leaking silicone oil – has been strongly recommended. Migration of the silicone oil has been reported to migrate in several locations. Its migration into the subconjunctival space usually results in the presentation of a cystic or shiny subconjunctival swelling [7,9,10]. Migration to the brain was also reported, with a variety of CNS-related symptoms such as: seizures, stroke, headache, and dizziness, while some being asymptomatic [3]. There were two previously reported cases -other than but identical to ours – with silicone oil migration to the upper eyelid causing blepharoptosis and xanthelasma-like lesions [6,11]. One of these was a case of a 48-year-old female, who presented with the complaint of blepharoptosis and xanthelasma-like lesion of her left periocular area in 2011. She had history of retinal detachment with silicone oil being used as a tamponade three months before her presentation. Histopathology of these lesions was consistent with silicone oil [6]. The second more recent paper in 2018 described a 68-year-old patient who presented with similar clinical features and history of retinal detachment procedure (with silicone oil) as well as Baerveldt implant two years prior to presentation 11]. Similarly, silicone migration into the periorbital tissue and orbit has been reported during evisceration in a patient with history of repeated retinal surgeries and also as chronic inflammation with silicone granuloma formation after the use of silicone implant in anophthalmic sockets [12,13].

Our case has been prepared and reported in line with the updated SCARE guidelines in: "The SCARE 2018 statement: Updating consensus Surgical CAse REport (SCARE) guidelines." International Journal of Surgery 2018; 60:132–136. The authors further stress that they have no financial disclosures related to their recommendations [14].

## 4 Conclusion

Silicone oil migration is not an uncommon phenomenon. Vitreoretinal surgeons should be alerted and advocated about possible extra ocular silicone oil migration. It is recommended to make sure that all sclerotomies are tightly sealed especially after the use of silicone oil. Some even recommended giving antiglaucoma medication after theses surgeries. Oculoplastic surgeons on the other hand should be oriented with the possible presentation of silicone granuloma as a xanthelasma-like skin lesion in the periocular area due to its migration.

## Conflict of interest

None.

## Source of funding

None.

## Ethical approval

IRB is not required for case reports. However, information was obtained and reported in a manner that was compliant with the standards set forth by the Health Insurance Portability and Accountability Act, and the Declaration of Helsinki as amended in 2013.

## Consent

General informed written consent was obtained from the patient including anonymous use of information for presentations and contributions.

## Author contribution

**Saleh Hamad Alrashed:** Data collection, Data interpretation, Drafting the text.

**Hind Manaa Alkatan:** Study design, Writing the paper.

**Adel H. Alsuhaibani:** Supervision.

## Registration of research studies

Not applicable.

## Guarantor

Hind M. Alkatan.

## Provenance and peer review

Not commissioned, externally peer-reviewed.
